# Birth Weight and the Risk of Cardiovascular Outcomes: A Report From the Large Population-Based UK Biobank Cohort Study

**DOI:** 10.3389/fcvm.2022.827491

**Published:** 2022-03-24

**Authors:** Xin Huang, Jun Liu, Lu Qi, Jonathan D. Adachi, Jing Wu, Ziyi Li, Qiong Meng, Guowei Li, Gregory Y. H. Lip

**Affiliations:** ^1^Center for Clinical Epidemiology and Methodology (CCEM), Guangdong Second Provincial General Hospital, Guangzhou, China; ^2^Department of Cardiology, Guangdong Second Provincial General Hospital, Guangzhou, China; ^3^Department of Epidemiology, School of Public Health and Tropical Medicine, Tulane University, New Orleans, LA, United States; ^4^Department of Medicine, McMaster University, Hamilton, ON, Canada; ^5^Key Laboratory of Environment and Health, Ministry of Education, Department of Epidemiology and Biostatistics, School of Public Health, Tongji Medical College, Huazhong University of Science and Technology, Wuhan, China; ^6^Department of Pediatrics, Guangdong Second Provincial General Hospital, Guangzhou, China; ^7^Department of Health Research Methods, Evidence, and Impact (HEI), McMaster University, Hamilton, ON, Canada; ^8^Liverpool Centre for Cardiovascular Science, University of Liverpool, Liverpool, United Kingdom

**Keywords:** epidemiology, birth weight, cardiovascular disease, association, cohort

## Abstract

**Background:**

Birth weight has been reported to be associated with the risk of incident cardiovascular disease (CVD); however, the relationship remains inconclusive. Here, we aimed to prospectively assess the associations between birth weight and CVD risk using the data from UK Biobank, a large-scale, prospective cohort study.

**Methods:**

We included 270,297 participants who were free of CVD at baseline and reported their birth weight for analyses. The primary outcome was incident CVD. Hazard ratios (HRs) and 95% confidence intervals (CIs) for outcomes were calculated using Cox proportional hazards models adjusted for potential confounding variables.

**Results:**

During a median follow-up of 8.07 years (IQR: 7.4–8.7 years), 10,719 incident CVD events were recorded. The HRs for low birth weight vs. normal birth weight (2.5–4.0 kg) were 1.23 (95% CI: 1.09–1.38) for risk of incident CVD, 1.52 (95% CI: 1.18–1.95) for stroke, 1.33 (95% CI: 1.07–1.64) for myocardial infarction, and 1.15 (95% CI: 1.01–1.32) for CHD. For the ones with low birth weight, the risk of CVD is reduced by 11% for every kilogram of birth weight gain. The association of low birth weight with CVD was stronger among those younger than 55 years (*p* = 0.001). No association between high birth weight and risk of cardiovascular outcomes was found.

**Conclusion:**

Low birth weight was associated with an increased risk of cardiovascular events. These findings highlight the longstanding consequence of low birth weight on cardiovascular system.

## Introduction

Cardiovascular disease (CVD) remains the leading cause of death and morbidity in the world (1, 2) and is a major public healthcare burden with considerable associated costs (3). There is evidence suggesting that susceptibility to CVD may have etiological origins *in utero* and in infancy (4). The “fetal origins” hypothesis proposes that a baby’s nourishment before birth and during infancy, as manifested in patterns of fetal and infant growth, may have long term consequences for physiological function and risk for adult disease (5). This may be due to an effect of prenatal growth on the pathogenesis of early disease, by “programming” the development of risk factors (e.g., insulin/glucose metabolism, hypertension, and lipid metabolism) (5, 6) or due to altered “stability” of established physiological conditions (6).

Birth weight is one of the most important surrogate measures of intrauterine growth and the prenatal environment, normally ranging from 2.5 to 4.0 kg (7). In 1989, birth weight was first reported to be associated with death rate from ischemic heart disease during adulthood (8). Since then, the associations between birth weight and risk of CVD [e.g., myocardial infarction, coronary heart disease (CHD), and stroke] has been explored in several independent cohorts (9). However, these studies were mainly focused on CHD and often single-sex (male or female), reported inconclusive findings (9), and their sample sizes were frequently limited yielding insufficient statistical power (10).

In this prospective cohort study, we examined the association between birth weight and risk of cardiovascular outcomes based on data from a large population-based cohort study. This study could provide information on clarification of the relationship between birth weight and cardiovascular outcomes.

## Materials and Methods

### Study Population

The UK Biobank is a large-scale, population-based prospective cohort study designed to improve the prevention, diagnosis and treatment of a wide range of diseases and to promote health throughout society (11). Between 2006 and 2010, the UK Biobank recruited over 500,000 men and women aged 40–70 years from across the UK. Participants provided electronic informed consent, completed touch screen questionnaires and face-to-face interviews and provided biological samples including samples of blood, urine and saliva. The UK Biobank study was approved by the National Health Service’s National Research Ethics Service and participants provided written informed consent.

### Study Endpoints

The primary outcome was a composite of incident CVD events including myocardial infarction, CHD and stroke. Secondary outcomes were stroke (ischemic and hemorrhagic stroke), myocardial infarction, CHD, CVD mortality, and all-cause mortality. Outcome adjudication involved hospital admission data or death records after birth. The hospital admission data were identified by linking to the Scottish Morbidity Records for participants from Scotland and health episode statistics for participants from England and Wales (11). Additionally, the death records were identified using death registries of the National Health Service Information Centers (11). The hospital registry-based follow-up ended on 31st March 2017, in England; 31st October 2016, in Scotland; and 29th February 2016, in Wales. At the time of analysis, mortality data were available up to 14 February 2018 for England and Wales, and 1 January 2017 for Scotland. The participants were followed up from the date of recruitment (between 2006 and 2010) to the date of death or the end of follow-up, whichever occurred first. We defined cardiovascular events according to a hospital admission or death with the following ICD-9 and/or ICD-10 (International Classification of Diseases, 9th revision and 10th revision) codes: CVD codes ICD-9 410-414 and ICD-10 I20-I25, stroke codes ICD-9 430-434,436 and ICD-10 I60-I64, and CVD death codes ICD-10 I00-I99. Ischemic stroke was defined by ICD-9 codes 433–434, and ICD-10 code I63, while hemorrhagic stroke was defined by ICD-9 codes 430–432 and ICD-10 codes I60-I62. Myocardial infarction was defined as ICD-9 codes 410–412, and ICD-10 code I21, I22, I23, I24.1, I25.2. CVD death was defined as ICD-10 codes I00-I99.

### Ascertainment of Variables

At baseline participants were asked to enter their own birth-weight. High birth weight implied growth beyond an absolute birth weight of 4.0 kg, while low birth weight was generally defined as any birth below 2.5 kg (7).

A baseline touch screen questionnaire was used to assess several potential confounders as follow. Sociodemographic characteristics involved age, sex, ethnicity, Townsend Deprivation Index (TDI) and education. Lifestyle behaviors included body mass index (BMI), physical activity (MET minutes per week for moderate activity), smoking status, alcohol consumption, vegetable and fruit consumptions. Maternal smoking, breastfed as a baby, and part of multiple birth were included in early life exposures. Drug use and dietary supplementations included aspirin use, non-aspirin NSAIDs use, vitamin (multivitamin, folic acid, vitamin A, vitamin B, vitamin C, vitamin D, or vitamin E), mineral and other dietary supplementations (calcium, iron, zinc, selenium, or fish oil). Health conditions consisted of hypertension, diabetes and dyslipidemia. Dyslipidemia was defined as elevated total cholesterol [≥240 mg/dL (6.20 mmol/L)], LDL cholesterol [>160 mg/dL (4.13 mmol/L)], or triglyceride [>200 mg/dL (2.25 mmol/L)]; or reduced HDL cholesterol [<40 mg/dL (1.03 mmol/L)] (12); or current usage of lipid-lowing medication. Other health conditions were obtained by self-report results with augmentation of these data using ICD-10 from hospital records. Further details of these measurements are available on the UK Biobank.^1^

### Statistical Analysis

Baseline characteristics are presented as means (SD) for continuous variables and as number (%) for categorical variables. We used ANOVA or chi-square tests to examine participant characteristics according to whether participants were normal, low or high birth weight at baseline. Cox proportional hazards models were conducted to estimate hazard ratios (HR) and 95% confidence intervals (95% CI) for associations of birth weight with risk of cardiovascular outcomes. We ran two models including the basic model (model 1) that was adjusted for age and sex, and the fully adjusted model (model 2) that was further adjusted for ethnicity (white or others), TDI, education (degree or no degree), BMI, physical activity (<500 or ≥500 MET-min/week), smoking status (never, former or current), alcohol consumption (never, former or current), vegetable and fruit consumptions (<2.0, 2.0–3.9 or ≥4.0 pieces/day or tablespoons/day), maternal smoking (yes or no), and following variables which were reported as present or absent, including breastfed as a baby, part of multiple birth, aspirin use, non-aspirin NSAID use, vitamin supplements use, mineral and other dietary supplements use, hypertension, diabetes, dyslipidemia. These factors were included because were well-known to be associated with the risk of developing CVD (13, 14), or commonly included in multivariate models for the outcomes (15, 16). Furthermore, with birth weight as a continuous exposure variable, we used restricted cubic splines with five knots at 5th, 27.5th, 50th, 72.5th, and 95th percentiles to evaluate the potential non-linear effect of birth weight on CVD incidence in the fully adjusted model (17).

We conducted predefined stratified analyses to assess potential effect modifications by the following factors: sex (male vs. female), age (<55 vs. ≥55 years), ethnicity (white vs. others), obesity (yes vs. no), physical activity (<500 vs. ≥500 MET-min/week), and following factors which were reported as present or absent, including hypertension, diabetes, dyslipidemia, aspirin use, non-aspirin NSAID use. We tested potential effect modifications by modeling the cross-product term of the stratifying variable with birth weight in the fully adjusted model.

We also conducted several sensitivity analyses to explore the robustness of our main results by (1) excluding the participants with family history of CVD, (2) performing a competing risk analysis that took all-cause death as a competing event for CVD, and (3) performing a model adjusted for the propensity score, where the propensity score was calculated by a logistic regression that included the aforementioned baseline covariates for birth weight. Besides, considering that chronic diseases (i.e., diabetes, hypertension, and dyslipidemia) may be a link in the chain of CVD etiology (18–20), we further performed a sensitivity analysis by excluding these covariates about health condition in the fully adjusted model.

We used the SAS (version 9.2; SAS Institute Inc, NC, USA), IBM SPSS software (version 22.0; SPSS Inc., Chicago, IL, United States) and R (version 3.5.1; R Foundation for Statistical Computing, Vienna, Austria) for analyses in this study. We considered a *P*-value less than 0.05 (two-sided) to be statistically significant.

## Results

### Baseline Participant Characteristics

We included 280,252 participants who had data on the birth weight, and excluded 9,276 with CVD at baseline and 679 who subsequently withdrew from the study. Finally, our analysis included a total of 270,297 participants (Supplementary Figure 1). Baseline characteristics of the included participants according to birth weight categories are shown in Table 1. Overall, 3.2 and 13.7% of the study population reported low and high birth weight at baseline, respectively. Compared to the participants with normal birth weight, those with low birth weight were older, more likely to be females, current smokers, non-breastfed as a baby, part of multiple birth and exposed to maternal smoking *in utero*. They also had a higher prevalence of hypertension, diabetes mellitus and dyslipidemia. When compared with participants with normal birth weight, those with high birth weight were older, more likely to be males, current smokers, former drinkers and breastfed as a baby. Baseline characteristics of participants (*n* = 232,196) excluded from our analysis are summarized in Supplementary Table 1, showing that they had similar characteristics to the participants included in this study.

**TABLE 1:** Baseline characteristics of study participants.

Characteristics	Normal BW (2.5–4.0 kg)	Low BW (<2.5 kg)	High BW (>4.0 kg)	*P*-value
N	224,528 (83.1)	8,780 (3.2)	36,989 (13.7)	
Age, years	54.85 (8.07)	56.53 (7.98)	55.76 (8.18)	<0.001
Male	81,275 (36.2)	2,809 (32.0)	19,017 (51.4)	<0.001
Ethnicity				<0.001
White	217,037 (96.7)	8,463 (96.4)	35,942 (97.2)	
Others	6,826 (3.0)	284 (3.2)	932 (2.5)	
TDI	–1.53 (3.03)	–1.13 (3.21)	–1.46 (3.07)	<0.001
Qualification				<0.001
Degree	78,739 (35.1)	2,170 (24.7)	12,537 (33.9)	
No degree	145,789 (64.9)	6,610 (75.3)	24,452 (66.1)	
BMI, kg/m^2^	27.09 (4.82)	27.83 (5.20)	27.92 (4.90)	<0.001
Physical activity, MET-min/week				<0.001
<500	101,053 (45.0)	3,609 (41.1)	16,148 (43.7)	
≥500	82,918 (36.9)	3,189 (36.3)	14,220 (38.4)	
Smoking				<0.001
Never	129,910 (57.9)	5,160 (58.8)	18,965 (51.3)	
Former	72,063 (32.1)	2,607 (29.7)	13,770 (37.2)	
Current	21,797 (9.7)	975 (11.1)	4,094 (11.1)	
Drinking				<0.001
Never	7,927 (3.5)	475 (5.4)	1,200 (3.2)	
Former	7,092 (3.2)	357 (4.1)	1,316 (3.6)	
Current	209,182 (93.2)	7,928 (90.3)	34,421 (93.1)	
Vegetable consumption, tablespoons/day				0.003
<2.0	10,603 (4.7)	485 (5.5)	1,807 (4.9)	
2.0–3.9	62,999 (28.1)	2,381 (27.1)	1,0265 (27.8)	
≥4.0	150,800 (67.2)	5,909 (67.3)	24,895 (67.3)	
Fruit consumption, pieces/day				<0.001
<2.0	58,607 (26.1)	2,446 (27.9)	9,853 (26.6)	
2.0–3.9	93,292 (41.6)	3,495 (39.8)	14,958 (40.4)	
≥4.0	72,503 (32.3)	2,834 (32.3)	12,156 (32.9)	
Early life exposure				<0.001
Maternal smoking	58,087 (25.9)	2,936 (33.4)	8,496 (23.0)	
Breastfed as a baby	141,397 (63.0)	3,434 (39.1)	23,722 (64.1)	
Part of multiple birth	5,256 (2.3)	2,004 (22.8)	130 (0.4)	
Supplement and medication use				
Vitamin	73,309 (32.7)	2,879 (32.8)	11,653 (31.5)	<0.001
Mineral and other dietary supplements	95,756 (42.6)	3,808 (43.4)	15,698 (42.4)	0.238
Aspirin	23,421 (10.4)	1,212 (13.8)	4,357 (11.8)	<0.001
Non-aspirin NSAIDs	75,354 (33.6)	3,217 (36.6)	12,069 (32.6)	<0.001
Health conditions				
Hypertension	109,816 (48.9)	5,234 (59.6)	18,214 (49.2)	<0.001
Diabetes	8,414 (3.7)	609 (6.9)	1,365 (3.7)	<0.001
Dyslipidemia	130,882 (58.3)	5,702 (64.9)	21,751 (58.8)	<0.001

*Values are presented as means (SD) for continuous variables and as number (%) for categorical variables. BW, birth weight; TDI, Townsend Deprivation Index; BMI, body mass index; NSAID, non-steroidal anti-inflammatory drug.*

### Association of Birth Weight and Cardiovascular Disease

A total of 10,719 cardiovascular events were observed during a median follow-up of 8.07 years (IQR: 7.4–8.7 years). Table 2 displays the association between low birth weight and incidence of CVD. We found significant associations between low birth weight and risk of overall cardiovascular events, stroke, myocardial infarction and CHD in model 1 adjusted for age and sex, with all *p*-values < 0.001.

**TABLE 2:** Association between low birth weight and risk of cardiovascular outcomes.

	Cases (incidence/1,000 person years)		Model 1†		Model 2^‡^			
Outcomes	Normal BW	Low BW	HR (95% CI)	*P*-value	HR (95% CI)	*P*-value	Per additional^‡^	*P*-value
CVD events	8,534 (4.83)	503 (7.37)	1.42 (1.30–1.54)	<0.001	1.23 (1.09–1.38)	0.001	0.89 (0.85–0.94)	<0.001
Stroke	1,905 (1.06)	120 (1.71)	1.45 (1.21–1.75)	<0.001	1.52 (1.18–1.95)	0.001	0.85 (0.76–0.94)	0.003
Ischemic stroke	1,198 (0.67)	74 (1.05)	1.42 (1.12–1.79)	0.004	1.37 (0.99–1.91)	0.061	0.89 (0.78–1.02)	0.10
Hemorrhagic stroke	593 (0.33)	35 (0.50)	1.38 (0.98–1.94)	0.067	1.69 (1.11–2.58)	0.014	0.80 (0.66–0.96)	0.016
Myocardial infarction	2,957 (1.65)	180 (2.58)	1.46 (1.26–1.70)	<0.001	1.33 (1.07–1.64)	0.009	0.90 (0.82–0.98)	0.013
CHD	10,170 (5.74)	605 (8.82)	1.40 (1.28–1.54)	<0.001	1.15 (1.01–1.32)	0.032	0.90 (0.86–0.95)	<0.001
CVD mortality	735 (0.37)	42 (0.54)	1.28 (0.98–1.66)	0.065	1.17 (0.80–1.72)	0.42	0.92 (0.79–1.06)	0.26
All-cause mortality	6,504 (3.26)	331 (4.25)	1.16 (1.04–1.29)	0.009	1.03 (0.87–1.21)	0.75	0.96 (0.90–1.02)	0.17

*CVD, cardiovascular disease; CHD, coronary heart disease; BW, birth weight; HR, hazard ratio; CI, confidence interval.*

*^†^Model 1: adjusted for age and sex.*

*^‡^Model 2: additionally adjusted for ethnicity, TDI, education, BMI, physical activity, smoking status, alcohol consumption, vegetable and fruit consumptions, maternal smoking, breastfed as a baby, part of multiple birth, aspirin use, non-aspirin NSAID use, vitamin, mineral and other dietary supplement use, hypertension, diabetes, dyslipidemia.*

In the multivariable analyses, low birth weight remained significantly related with increased risk of CVD (HR = 1.23, 95% CI 1.09–1.38), stroke (HR = 1.52, 95% CI 1.18–1.95), hemorrhagic stroke (HR = 1.69, 95% CI 1.11–2.58), myocardial infarction (HR = 1.33, 95% CI 1.07–1.64), and CHD (HR = 1.15, 95% CI 1.01–1.32). No association between macrosomia and risk of cardiovascular outcomes was found (Table 2). Neither low birth weight nor macrosomia was significantly related with risk of CVD mortality or all-cause mortality (*p*-values > 0.05; Tables 2, 3 and Supplementary Table 2).

**TABLE 3:** Association between high birth weight and risk of cardiovascular outcomes.

	Cases (incidence/1,000 person years)		Model 1†		Model 2^‡^			
Outcomes	Normal BW	High BW	HR (95% CI)	*P*-value	HR (95% CI)	*P*-value	Per additional^‡^	*P*-value
CVD events	8,534 (4.83)	1,682 (4.69)	0.98 (0.94–1.03)	0.46	0.98 (0.92–1.05)	0.61	0.96 (0.92–1.00)	0.046
Stroke	1,905 (1.06)	346 (0.95)	0.95 (0.84–1.06)	0.35	1.00 (0.86–1.16)	0.99	0.96 (0.88–1.06)	0.42
Ischemic stroke	1,198 (0.67)	225 (0.61)	0.95 (0.83–1.10)	0.50	1.03 (0.86–1.24)	0.75	0.99 (0.89–1.11)	0.92
Hemorrhagic stroke	593 (0.33)	103 (0.28)	0.96 (0.78–1.19)	0.72	0.91 (0.69–1.20)	0.49	0.89 (0.75–1.05)	0.17
Myocardial infarction	2,957 (1.65)	595 (1.63)	0.97 (0.88–1.06)	0.45	0.96 (0.86–1.08)	0.50	0.95 (0.88–1.02)	0.15
CHD	10,170 (5.74)	1,979 (5.51)	0.99 (0.94–1.04)	0.59	0.98 (0.92–1.05)	0.59	0.96 (0.92–1.00)	0.037
CVD mortality	735 (0.37)	160 (0.39)	1.08 (0.94–1.25)	0.26	0.94 (0.78–1.15)	0.55	0.94 (0.84–1.06)	0.34
All-cause mortality	6,504 (3.26)	1,307 (3.22)	1.05 (0.99–1.12)	0.080	1.01 (0.94–1.10)	0.75	0.99 (0.94–1.04)	0.66

*CVD, cardiovascular disease; CHD, coronary heart disease; BW, birth weight; HR, hazard ratio; CI, confidence interval.*

*^†^Model 1: adjusted for age and sex.*

*^‡^Model 2: additionally adjusted for ethnicity, TDI, education, BMI, physical activity, smoking status, alcohol consumption, vegetable and fruit consumptions, maternal smoking, breastfed as a baby, part of multiple birth, aspirin use, non-aspirin NSAID use, vitamin, mineral and other dietary supplement use, hypertension, diabetes, dyslipidemia.*

Besides, as shown in Table 2, for the ones with low birth weight, the risk of CVD is reduced by 11% for every kilogram of birth weight gain (HR = 0.89, 95% CI 0.85–0.94), stroke by 15% (HR = 0.85, 95% CI 0.76–0.94), and CHD by 10% (HR = 0.90, 95% CI 0.86–0.95). For the ones with high birth weight, the risks of CVD and CHD are reduced by 4% for every kilogram of birth weight gain, (HR = 0.96, 95% CI 0.92–1.00).

**FIGURE 1:**
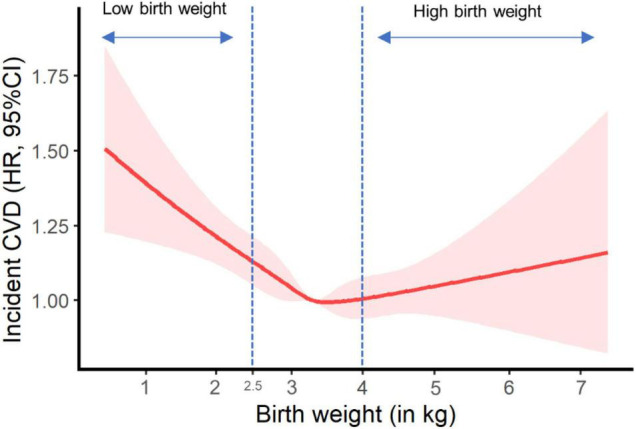
Plot of incident cardiovascular disease risk against birth weight. HR, hazard ratio; CI, confidence interval; NSAID, non-steroidal anti-inflammatory drug. Results were adjusted for age, sex, ethnicity, Townsend Deprivation Index, education, BMI, physical activity, smoking status, alcohol consumption, vegetable and fruit consumptions, maternal smoking, breastfed as a baby, part of multiple birth, aspirin use, non-aspirin NSAID use, vitamin, mineral and other dietary supplement use, hypertension, diabetes, dyslipidemia.

Figure 1 shows the potential non-linear effect of birth weight (as a continuous exposure variable) on CVD incidence in the fully adjusted model, with the lowest CVD risk falling in normal birth weight (p for non-line = 0.002).

### Subgroup and Sensitivity Analyses

**FIGURE 2:**
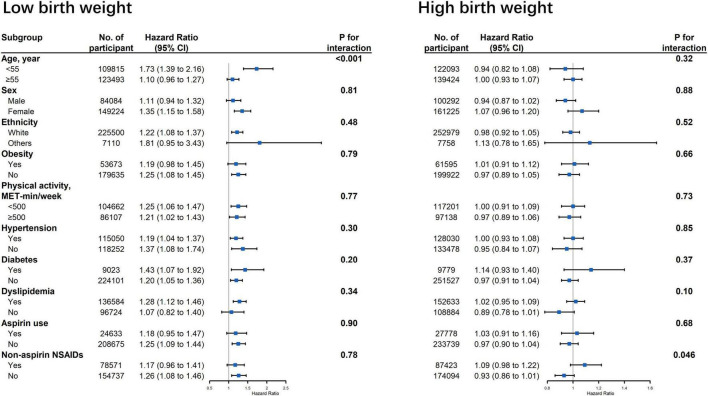
Association between birth weight and risk of cardiovascular disease stratified by potential effect modifications. CI, confidence interval; NSAID, non-steroidal anti-inflammatory drug. Results were adjusted for age, sex, ethnicity, Townsend Deprivation Index, education, BMI, physical activity, smoking status, alcohol consumption, vegetable and fruit consumptions, maternal smoking, breastfed as a baby, part of multiple birth, aspirin use, non-aspirin NSAID use, vitamin, mineral and other dietary supplement use, hypertension, diabetes, dyslipidemia.

Figure 2 displays the associations between birth weight and risk of cardiovascular events stratified by potential effect modifications. We observed a significant interaction between low birth weight and age on CVD risk (Figure 2, p for interaction < 0.001). The association between low birth weight and CVD risk was stronger among participants younger than 55 years old when compared with the older participants (HR: 1.73 vs. 1.10). The association between low birth weight and CVD outcomes were not modified by other risk factors, including gender, ethnicity, obesity, physical activity, hypertension, diabetes, dyslipidemia, aspirin, and non-aspirin NSAID use. No interaction was observed between high birth weight and all the aforementioned risk factors (Figure 2).

In the sensitivity analyses, the associations between low birth weight and CVD outcomes did not change appreciably when we excluded the participants with family history of CVD; when we performed a competing risk analysis that took all-cause death as a competing event for CVD; when we performed a model adjusted for the propensity score, and when we excluded the covariates about health condition in the fully adjusted model (Table 4).

**TABLE 4:** Sensitivity analysis of associations between birth weight and risk of cardiovascular outcomes.

Cardiovascular events	Normal BW	Low BW	Model 2^‡^		High BW	Model 2^‡^	
			HR (95% CI)	*P*-value		HR (95% CI)	*P*-value
When we excluded the participants with family history of CVD	7,530	451	1.19 (1.04–1.37)	0.012	1,495 (4.2)	0.99 (0.92–1.07)	0.83
When we performed a competing risk analysis that took all-cause death as a competing event for CVD	10,170	605	1.29 (1.14–1.46)	<0.001	1,979 (5.4)	0.98 (0.92–1.05)	0.55
When we performed a model adjusted for the propensity score^†^	10,170	605	1.26 (1.12–1.42)	<0.001	1,979 (5.4)	0.99 (0.93–1.06)	0.81
When we excluded the covariates about health condition in the fully adjusted model	101,70	605	1.29 (1.14–1.45)	<0.001	1,979 (5.4)	0.95 (0.89–1.01)	0.11

*CVD, cardiovascular disease; BW, birth weight; HR, hazard ratio; CI, confidence interval.*

*^†^The propensity score was calculated by a logistic regression that included the aforementioned baseline covariates for birth weight, and the model was adjusted for the propensity score.*

*^‡^Model 2: adjusted for age, sex, ethnicity, TDI, education, BMI, physical activity, smoking status, alcohol consumption, vegetable and fruit consumptions, maternal smoking, breastfed as a baby, part of multiple birth, aspirin use, non-aspirin NSAID use, vitamin, mineral and other dietary supplement use, hypertension, diabetes, dyslipidemia.*

## Discussion

In this large cohort study involving 270,297 individuals, we found that low birth weight was significantly associated with a 23% higher risk of overall cardiovascular events and a 15–59% higher risk of individual cardiovascular events (stroke, myocardial infarction, and CHD). For the ones with low birth weight, the risk of CVD is reduced by 11% for every kilogram of birth weight gain. The associations were independent of sociodemographic characteristics, lifestyle behaviors, early life exposures, medication use and dietary supplementations, and health conditions. The findings remained robust on multiple sensitivity analyses. Additionally, the associations between low birth weight and CVD were significantly modified by age. No association between high birth weight and cardiovascular outcomes was observed.

### Comparison With Other Studies

The association between fetal growth and cardiovascular outcomes has been extensively studied in many prospective cohort studies. Birth weight has been considered as a proxy measure of fetal growth, and a combined measure of at least two important components including fetal growth rate and gestational age at birth (21). The majority of but not all prior studies (9) of birth weight and risk of CVD reported an inverse association, with the strength of associations varying between studies. Moreover, previous studies mainly focused on CHD and were conducted in participants born in early twentieth century with limited sample sizes.

In our study, low birth weight was found to relate with a higher risk of CVD (e.g., CHD and stroke) in more than a quarter of a million UK participants born during 1934–1970. Besides, we observed a U-shaped effect of continuous birth weight on CVD incidence, with the lowest CVD risk falling in normal birth weight and the highest risk in low birth weight. This further supports the relationship between low birth weight and elevated risk of CVD as reported in our main analyses. However, we did not observe a significant association between birth weight and CVD mortality or death from individual cardiovascular outcomes, similar to other publications (9, 10). This might imply that low birth weight was related with the susceptibility of non-fatal CVD, rather than fatal CVD. Moreover, the risks of incident CVD and CVD mortality were not significantly linked to high birth weight (> 4.0 kg), consistent with previous studies (9, 10). The “growth acceleration” hypothesis by Singhal and Lucas (22) may help interpret the difference in the associations of CVD risk with low and high birth weight. The hypothesis raises the concept that relative slower infant growth benefits later CVD and its risk factors (22), in which the catch-up growth for those with low birth weight may lead to relatively higher postnatal growth and consequently accelerate the development of later CVD. As a proxy measure of fetal growth, more clinical attention should perhaps be needed for low birth weight rather than high birth weight in CVD prevention.

### Underlying Mechanisms of Low Birth Weight in Relation to Cardiovascular Disease

The “fetal origins” hypothesis proposes that impairment of intrauterine growth may have long term consequences for physiological function and risk for adult disease (23). Adverse intrauterine environmental factors that results in low birth weight may “program” permanent changes in organ development and metabolism (particularly those involved with insulin/glucose metabolism, hypertension and lipid metabolism), leading to future adult disease (6). Reduced growth *in utero* has also been shown to associate with an increased risk of hypertension, non-insulin dependent diabetes and higher concentrations of low-density lipoprotein cholesterol in adult life (20, 24, 25). These findings have generated the hypothesis that CVD originates from prenatal programming whereby malnutrition during sensitive periods in early life permanently changes the body’s structure and physiology (26). Meanwhile, intrauterine conditions may cause life-long alterations of key hormonal axes such as the hypothalamic–pituitary–adrenal axis, predisposing to elevated blood pressure and impaired glucose tolerance (18, 19). The mechanisms underlying the inverse association between low birth weight and cardiovascular outcomes are unclear, but insulin resistance and its associated metabolic risk factors may be important.

The association between low birth weight and CVD risk supports the hypothesis that the disease originates through patterns of reduced fetal growth in which the brain is spared. The mechanism of brain-sparing involved redistribution of cardiac output to favor the brain at the expense of the trunk (27). The structure of fetal arteries adapts to and redistribution of cardiac output may therefore permanently change the structure of the major arteries. This may lead to persistently reduced elasticity and consequent raised blood pressure, a major risk factor for hemorrhagic stroke (28). Moreover, recent animal studies indicates that intrauterine conditions may have a profound impact on adult lifestyle behavior such as stress responses and level of physical activity that are likely to affect the incidence of CVD (19, 29, 30).

### Strengths and Limitations

This study used data from a large-scale prospective cohort to extensively investigate the association between birth weight and CVD. We performed rigorous analyses including multivariable models with careful adjustment for the covariates, subgroup and sensitivity analyses, which yielded robust and consistent findings. Other strengths included the large and nationally representative sample, a high follow-up rate, and the amount of information available. However, our study has several limitations. First, approximately only a half of the participants had data on birth weight that were self-reported. No validation of self-reported birth weights could be carried out in this cohort. However, in a number of studies self-reported birth weight has been found to correlate well with hospital records (correlation coefficients in the range of 0.64–0.86), though absolute levels of accuracy may be poor (31). This issue seems not to alter the overall association between birth weight and CVD, as reported by other previous studies (9, 10). Second, the mean age of the participants was 55 years old at baseline; consequently, survivor bias was unavoidable. Nevertheless, our stratified analyses indicated that the association of low birth weight and CVD was stronger in subjects younger than 55 years (Figure 2). We might therefore have underestimated the association between low birth weight and CVD risk to an unknown degree. Third, the information at baseline was limited, such as gestational age. Birth weight has been understood as a combined measure of postnatal nutrition and gestational age at birth. Therefore, we were not able to distinguish whether the association was from undernutrition or preterm birth. Also, the data of this study were still limited and the baseline information was mostly collected through self-reported questionnaires. Thus, information bias was inevitable, especially for data on maternal exposure and “catch-up growth” of neonates with low birth weight. Fourth, most of covariates were available only at baseline, so we did not use time varying covariates to capture changes in possible confounders over time.

## Conclusion

When compared with normal birth weight, low birth weight, but not macrosomia, was associated with an increased risk of cardiovascular events. These findings may inform CVD screening and primary prevention strategies for those with low birth weight.

## Data Availability Statement

The datasets presented in this study can be found in online repositories. The names of the repository/repositories and accession number(s) can be found below: UK Biobank (www.ukbiobank.ac.uk/).

## Ethics Statement

The studies involving human participants were reviewed and approved by the National Health Service’s National Research Ethics Service. The patients/participants provided their written informed consent to participate in this study.

## Author Contributions

XH, GWL, and QM conceived and designed the study. XH and GWL conducted the data analysis. XH drafted the manuscript. JL, LQ, JA, JW, ZL, QM, GWL, and GYHL participated in the interpretation of the results and critically revised the manuscript for important intellectual content. GWL was guarantor, had full access to all of the data in the study, took responsibility for the integrity of the data, and the accuracy of the data analysis. QM and GWL attested that all the listed authors meet authorship criteria and that no others meeting the criteria have been omitted. All authors contributed to data analysis, drafting or revising the article, gave final approval of the version to be published, and agreed to be accountable for all aspects of the work.

## Conflict of Interest

The authors declare that the research was conducted in the absence of any commercial or financial relationships that could be construed as a potential conflict of interest.

## Publisher’s Note

All claims expressed in this article are solely those of the authors and do not necessarily represent those of their affiliated organizations, or those of the publisher, the editors and the reviewers. Any product that may be evaluated in this article, or claim that may be made by its manufacturer, is not guaranteed or endorsed by the publisher.

## Footnotes

1
https://www.ukbiobank.ac.uk/
