# Supplementary material for: Birth Weight and the Risk of Cardiovascular Outcomes: A Report From the Large Population-Based UK Biobank Cohort Study
Source: Front Cardiovasc Med. 2022 Mar 24;9:827491. doi: 10.3389/fcvm.2022.827491 (PMC8987713; doi:10.3389/fcvm.2022.827491)
Supplement: Supplementary file 1 [file Data_Sheet_1.pdf]

Supplementary Materials

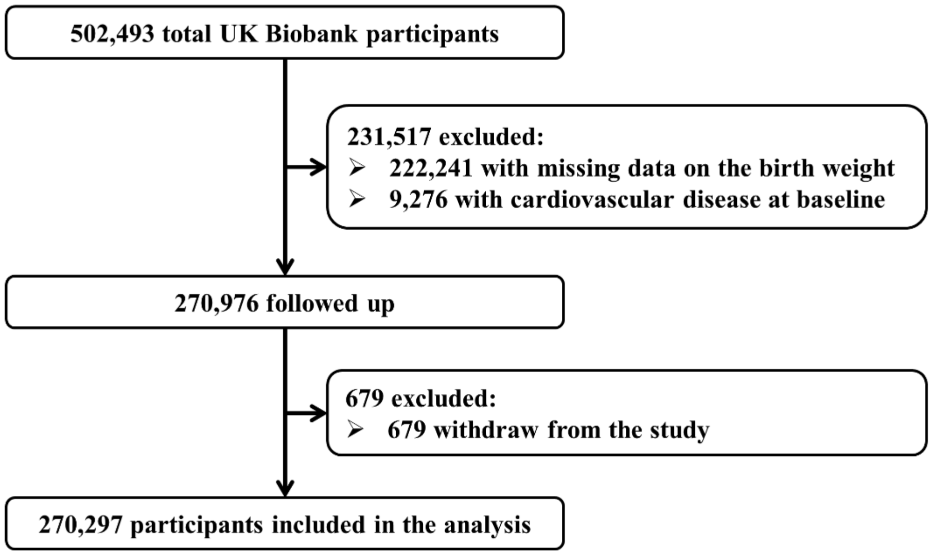

Supplementary Figure 1 Flow chart of participant selection in this study

Supplementary Table 1 Baseline characteristics of participants in UK biobank excluded from our analysis

Characteristics	Participants excluded
N	232196
Age, years	58.28 (7.74)
Male	126014 (54.3)
Ethnicity	
White	211296 (91.0)
Others	18996 (8.2)
Townsend Deprivation Index	-1.07 (3.30)
Qualification	
Degree	68191 (29.4)
No degree	158422 (68.2)
Body mass index, kg/m^2	27.67 (4.73)
Physical activity, MET-min/week	
< 500	98235 (42.3)
≥ 500	82999 (35.7)
Smoking	
Never	119481 (51.5)
Former	84611 (36.4)
Current	26111 (11.2)
Drinking	
Never	12783 (5.5)
Former	9337 (4.0)
Current	208821 (89.9)
Vegetable consumption, tablespoons/day	
< 2.0	14596 (6.3)
2.0-3.9	66673 (28.7)
≥ 4.0	150186 (64.7)
Fruit consumption, pieces/day	
< 2.0	67214 (28.9)
2.0-3.9	92280 (39.7)
≥ 4.0	71960 (31.0)
Early life exposure	
Maternal smoking	57104 (24.6)
Breastfed as a baby	109041 (47.0)
Part of multiple birth	3840 (1.7)
Supplement and medication use	
Vitamin	70078 (30.2)

Mineral and other dietary supplements	98523 (42.4)
Aspirin	47131 (20.3)
Non-aspirin NSAIDs	73419 (31.6)
Health conditions	
Hypertension	136951 (59.0)
Diabetes	16156 (7.0)
Dyslipidemia	154830 (66.7)

Values are presented as means (SD) for continuous variables and as number (%) for categorical variables.

Abbreviations: NSAID, non-steroidal anti-inflammatory drug.

Supplementary Table 2 Associations between birth weight and risk of CVD mortality by stroke, myocardial infarction and CHD

Outcomes	Normal BW	Low BW	Model 2‡		High BW	Model 2‡	
			HR (95% CI)	P_{value}		HR (95% CI)	P_{value}
Mortality from stroke	173	9	1.17 (0.50 to 2.70)	0.72	33	1.02 (0.65 to 1.58)	0.94
Ischemic stroke	17	0	-	-	4	0.54 (0.12 to 2.38)	0.42
Hemorrhagic stroke	111	7	1.47 (0.57 to 3.75)	0.42	24	1.42 (0.86 to 2.37)	0.17
Mortality from myocardial infarction	186	9	0.93 (0.37 to 2.33)	0.88	40	0.85 (0.55 to 1.30)	0.45
Mortality from CHD	478	25	1.12 (0.65 to 1.94)	0.68	107	0.87 (0.66 to 1.15)	0.33

Abbreviations: CHD, coronary heart disease; BW, birth weight; HR, hazard ratio; CI, confidence interval.

‡Model 2: adjusted for age, sex, ethnicity, TDI, education, BMI, physical activity, smoking status, alcohol consumption, vegetable and fruit consumptions, maternal smoking, breastfed as a baby, part of multiple birth, aspirin use, non-aspirin NSAID use, vitamin, mineral and other dietary supplement use, hypertension, diabetes, dyslipidemia.
